# Genome Sequences of Bacteriophages UPEC01, UPEC03, UPEC06, and UPEC07 Infecting Avian Pathogenic Escherichia coli

**DOI:** 10.1128/mra.00811-21

**Published:** 2022-03-09

**Authors:** George Kazibwe, Christian Ndekezi, Ruth Alinaitwe, Stephen Alafi, Ann Nanteza, Magambo Phillip Kimuda, Jesca Lukanga Nakavuma

**Affiliations:** a School of Biosecurity, Biotechnical and Laboratory Sciences, College of Veterinary Medicine, Animal Resources and Biosecurity, Makerere University, Kampala, Uganda; b Department of Biomolecular and Biolab Sciences, College of Veterinary Medicine, Animal Resources and Biosecurity, Makerere University, Kampala, Uganda; DOE Joint Genome Institute

## Abstract

Here, we present the genome sequences of four bacteriophages that infect avian pathogenic Escherichia coli. The phages were isolated from raw sewage in Kampala, Uganda. The genome sizes of the phages ranged between 143,140 bp and 178,307 bp, with an average G+C content of 41.25%.

## ANNOUNCEMENT

Phages infecting avian pathogenic Escherichia coli (APEC) have the potential to be applied as phage therapy in the management of avian colibacillosis, a devastating disease that is responsible for significant economic losses in the poultry industry (1). The emergence of multidrug-resistant pathogenic E. coli strains has sparked interest in the search for alternative control measures for bacterial pathogens, including, among others, the use of phages (2). In this study, whole-genome sequencing of bacteriophages was carried out to determine the genetic characteristics and the taxonomic identification or classification of these phages as part of a larger study aimed at identifying and establishing phage stocks that can be used to supplement the use of antibiotics in managing avian colibacillosis in Uganda.

The bacteriophages in this study were isolated from sewage at the National Water and Sewerage Corporation treatment plant (Kampala, Uganda). Several E. coli field isolates (Table 1) obtained from chicken droppings were used as isolation hosts for the phages following previously described methods (3). Briefly, 10 mL of raw sewage was centrifuged (10,000 × *g* for 10 min) to obtain a supernatant, which was added to 10 mL of 2× tryptic soy broth (TSB) containing 100 μL of overnight E. coli broth culture. The mixture was incubated (30°C for 48 h at 120 rpm) and centrifuged (7,000 rpm for 5 min at 4°C), and the supernatant was filtered (0.45 μm). The phage lysate obtained was plaque purified three times to produce a uniform phage stock. The isolated phages that could infect the APEC isolates from chickens that had died from colibacillosis were selected (4). Genomic DNA was extracted from the phages using 2% SDS and purified using a Qiagen Genomic-tip 100/G kit according to the manufacturer’s instructions.

**TABLE 1:** Features of Escherichia coli phages infecting APEC

Phage	Isolation host	Genome size (bp)	No. of ORFs	G+C content (%)	Family	Gene density (no. of genes/kb)	GenBank accession no.	SRA accession no.
UPEC01	S3Lug	169,801	307	40	*Myoviridae*	1.8	MW233709	SRX12305553
UPEC03	19-1331	178,307	279	44	*Myoviridae*	1.6	MW250785	SRX12305929
UPEC06	19-10951	143,140	308	41	*Myoviridae*	2.2	MW250786	SRX12306092
UPEC07	2	170,004	308	40	*Myoviridae*	1.8	MW250787	SRX12306233

Library preparation of the purified phage genomes was performed using the Nextera XT library preparation kit, and the libraries were sequenced using the Illumina MiSeq sequencing platform at Intellectual Ventures, USA, yielding 158,716, 149,362, 73,143, and 161,123 paired-end reads (151 bp in length) for phages UPEC01, UPEC03, UPEC06, and UPEC07, respectively. The sequenced reads were checked for quality using FastQC and MultiQC packages (5). The bad reads and adapter content were removed using the Trim Galore package with cutoff Phred scores of 30. The trimmed reads were then *de novo* assembled using SPAdes v3.11.1 (6). The genes of UPEC01, UPEC03, UPEC06, and UPEC07 were predicted from the genomes using PHANOTATE, which revealed 307, 279, 308, and 308 open reading frames (ORFs), respectively (7). The proteins encoded by these ORFs were functionally annotated using PANNZER2 (8), while PhageTerm was used to determine the phage genome termini (9). Unless otherwise indicated, all of the tools used for analysis were run using the default settings.

The phage genomes varied between 143,140 bp and 178,307 bp of double-stranded DNA (dsDNA), with an average density of 1.85 genes/kb and an average G+C content of 41.25% (Table 1). A BLASTn search (http://blast.ncbi.nlm.nih.gov) showed that UPEC01, UPEC03, UPEC06, and UPEC07 phages are similar to enterobacteria phage vB_EcoM_VR20 (GenBank accession number NC_28894), *Cronobacter* phage vB_CsaM_GAP161 (GenBank accession number NC_019398), *Cronobacter* phage PBES 02 (GenBank accession number NC_028672), and enterobacteria phage vB_EcoM_VR20 (GenBank accession number NC_028894), sharing 95%, 93.7%, 75%, and 89% nucleotide sequence identity, respectively. Analysis of proteins from UPEC01, UPEC03, UPEC06, and UPEC07 genomes further confirmed the relatedness to the aforementioned phages; of the predicted ORFs, 287, 271, 263, and 291 sequences, respectively, were functionally annotated. Among the putative proteins encoded by these ORFs are the terminase proteins involved in packaging of the phage genome into preformed empty capsids and the tail sheath proteins that facilitate entry of the phage DNA into the target host cell.

### Data availability.

The raw reads have been deposited in the NCBI Sequence Read Archive (SRA) under the BioProject accession number PRJNA765519 and the genome sequences in the NCBI GenBank database. The associated accession numbers are listed in Table 1.
